# Differences in Visuospatial Expertise between Skeet Shooting Athletes and Non-Athletes

**DOI:** 10.3390/ijerph18158147

**Published:** 2021-07-31

**Authors:** Henrique Nascimento, Cristina Alvarez-Peregrina, Clara Martinez-Perez, Miguel Ángel Sánchez-Tena

**Affiliations:** 1 ISEC Lisboa—Instituto de Educação e Ciência de Lisboa, 1750-179 Lisboa, Portugal; henrique.nascimento@iseclisboa.pt (H.N.); masancheztena@ucm.es (M.Á.S.-T.); 2 Faculty of Biomedical and Health Science, Universidad Europea de Madrid, 28670 Madrid, Spain; cristina.alvarez@universidadeuropea.es; 3 Department of Optometry and Vision, Faculty of Optics and Optometry, Universidad Complutense de Madrid, 28037 Madrid, Spain

**Keywords:** visual performance, sports vision, visuospatial expertise

## Abstract

Background: Sports vision is a specialisation of optometry whose objective is to improve and preserve visual function to increase sports performance. The main objective of the present study was to compare the visual expertise of non-athletes to skeet shooting athletes. Methods: Participants underwent an optometric assessment in which all those with severe deviations from normal vision, after compensating for visual abnormalities, were eliminated. After that, the following six visuospatial components were measured: hand–eye coordination, peripheral awareness, fixation disparity, saccadic eye movements, speed of recognition and visual memory. To measure the aforementioned components, the following tests were used: directional arrows, similar and different characters, the dichromatic disparity test, character marking, a tachistoscopic test and tic-tac-toe using COI-vision software. Results: Skeet shooting athletes performed significatively better (*p* ≤ 0.05) in two out of the six tests: hand–eye coordination and visual memory. Conclusions: Although this study does not support the theory that athletes—in this case, skeet shooting athletes—perform significantly better in most components of the visuospatial tests, visual memory and hand–eye coordination are exceptions. To be more accurate in distinguishing between athletes and non-athletes, specific testing methods that can be used by a wide variety of disciplines should be developed. Training the weakest aspects of athletes can improve their sports performance.

## 1. Introduction

Sports vision is a relatively new field. It is crucial that all athletes are aware of the importance of their visual system and the impact that it can have on their sports performance. In some countries, such as the United States, visual training is routinely performed on all athletes; in other countries, it remains an unknown discipline [1]. The needs imposed on the visual system during sporting performance are amongst the strictest of any activity. Since the ability of an athlete to perform sports tasks is influenced by their vision, research has been carried out concerning the relationship between motor skills and vision. The existing literature on sports vision demonstrates that athletes have better visual skills than their non-athletic peers [2,3,4,5,6,7]; that those visual skills differ from different sports disciplines; [7,8] that vision skills can be improved through visual training [9,10]; and that improved visual abilities can be translated into a better sports performance [3,11].

Regarding the specific aspect of shooting, the main scientific references are the studies carried out by Howard [12] and Gregg [13], in which both emphasise the importance of visual acuity during shooting and describe some rules of refraction. The discipline under analysis, skeet shooting, is not well known or advertised. In Portugal, it is affiliated with the Portuguese Shooting Federation. Often called “trap”, this Olympic sport debuted in the Olympic Games of 1900, and it remains an Olympic sport today. It is considered the most difficult discipline in gun shooting sports. The objective is to shoot at a specific number of disc-shaped targets. These discs, which are 11 cm in diameter and made of pitch and limestone, are thrown from five different positions from a pit situated 15 m from the shooter. In each of these five positions there are three machines that launch the discs at a 45° angle and at a speed of 100 Km/h. The shooter does not know from which position the disc will come out, and the longer it takes them to shoot, the farther away the disc will be. Ideally, the first shot is fired within 0.7 s and the following shot within 0.9 s, requiring shooters to possess good reflexes. In contrast to other shooting disciplines such as bullseye pistol shooting, the relaxation of accommodation is key for its performance [14,15,16]. In archery, a profile of high values of distance visual acuity has been established, [17] as well as a relationship with auditory biofeedback that causes significant improvements in the performance of this discipline. It has been proved that visual training programmes can improve the visual function. The improvements were statistically significant in the following variables: distance strength, distance vergence recovery points, binocular analytical range, negative relative accommodation, saccadic fixations, and ease of near–far accommodation [18].

Improving visual performance through visual training becomes crucial when practicing skeet shooting, as the shooter has both eyes open while shooting. The relationship between the eyes is important. The visual acuity of both the dominant and non-dominant eyes should be as symmetrical as possible in both far and near accommodation.

The general physical condition of the shooting participants is also crucial. Several studies observe a relationship between certain physical skills and performance in this sport. It is suggested that physical factors such as cardiovascular health, respiratory resistance, stamina, strength, flexibility, power, coordination and/or agility, balance and precision contribute to accuracy and precision in shooting [19]. Factors such as grip stability [19,20,21,22], aiming accuracy, shot accuracy, firing time [23] and postural balance may also be relevant to shooting accuracy [20,21,22,24,25].

Shooting sports, such as trap, double trap and skeet, are physical activities that are influenced by both dynamic and static demands. However, these demands are rarely taken into account when prescribing current sports shooting techniques. Rifle shooting is defined as a static sport based on the limited movement required during execution [26]. Rifle targets are placed in a stationary or static position. The shooter remains static in one position until the round is fired and the target is intercepted. This is because minimal movement of the body and barrel of the rifle during firing contributes to greater precision, greater location of the shot and greater firing success of the rifle [27]. In contrast, shotgun shooting is a dynamic sport that requires precise rhythmic movement of the upper extremities, lower extremities and torso to successfully intercept a moving/dynamic target [28].

Shooting sports require great spatial awareness, analytical skills and resistance to fatigue. Highly experienced shooters can strike targets that are less than 0.1° of sight distance, with an initial speed of 33 m/s and, in some disciplines (such as trap), moving in unpredictable directions from unpredictable locations.

However, scientific evidence on basic visual–motor skills in shooters is conflicting. In 1999, Abernethy and Neal found no differences between a group of athletes and a control group through rapid tachistoscopic detection and detection of coincidence time [29]. Conversely, Czigler, Balazs and Lenart (1998) [30] found that the shooters were faster (48 ms) and more accurate than the control group in a task that required rapid information processing and simultaneous evaluation of visual characteristics.

It was not until 2010 that Causer et al. [28] first analysed the quiet eye in the sport of shotgun shooting. In the past, all investigations had focused primarily on self-paced static aiming tasks or tasks in which objects were brought closer to the participant. These authors conducted a comprehensive investigation of the sub-disciplines of shotgun shooting to assess gaze behaviour and kinematic differences between elite and sub-elite shooters, as well as successful and unsuccessful shots. They found that elite athletes across all three disciplines exhibited a longer quiet duration and earlier start compared to their sub-elite counterparts. Participants exhibited longer QE durations and earlier QE onset on successful shots compared to missed shots. Therefore, elite shooters demonstrated a more efficient pistol barrel movement, characterised by a smaller pistol barrel offset and a more efficient timing strategy.

In terms of balance and body stability, sports shooting involves many factors, such as concentration and cognition. Accuracy when aiming and shooting represents 81% of the shooting performance. Recent studies prove that body stability has an insignificant influence on the shooting performance [22]. Physical and mental performance may vary in relation to the circadian cycle, which is ruled by internal and external stimuli. The predominant external stimulus of the circadian cycle is light, which acts through the ganglion cells of the retina [31]. New studies question the prerequisite of retinal light transduction, showing an improvement in reaction time through a non-retinal transcranial pathway [32,33].

Olympic shooting requires a high level of accuracy, consistency and stability from the shooter [34]. In fact, the levels of accuracy are so high that angular mistakes over 0.016° while shooting a shotgun [35] or 0.066° while shooting a pistol [36] can prevent a shooter from achieving the maximum target shooting score. A number of studies also indicate the importance of psychological training [37,38] in order to improve performance so that shooters can achieve their highest levels.

Based on these data, we were interested in studying the relationship between the ability to perform certain tasks, coordination and speed of reaction, and we aimed to determine whether this relationship manifests differently in athletes and in non-athletes with the same basic visual skills, which would be established by predefined criteria for the visual analysis, having been proven by previous studies to be the most important factor for sports shooters. If the vision conditions are the same for athletes and non-athletes, and the values of the coordination and ability tests are identical, the results may indicate that by participating in visual training programmes, athletes can improve their sports performance, as various studies show. In addition, this may indicate that a compensated visual acuity is vital for the practice of any shooting discipline.

## 2. Materials and Methods

### 2.1. Data Collection

A cross-sectional design study was conducted between November 2018 and June 2019 at the High-Performance Centre in Sports Vision of ISEC Lisbon (Lumiar, Lisbon, Portugal). Twenty elite skeet shooting athletes and one hundred non-sporting individuals were randomly selected within the same age range. For the analysis of the visual function, the COI-Sport digital system (Madrid, Spain) was used, where the tests that analyse the most relevant visual skills for skeet shooting were selected according to the existing bibliography [39,40].

Hand–eye coordination: The directional arrows test was used. In this test, arrows are presented sequentially and within a fraction of second, pointing in different directions. The participant must touch the arrow directly on the screen before the arrow changes its direction. The speed of execution is important, as the number of correct answers and the execution time are recorded.

Peripheral awareness: The same characters test was used. In this test, one out of a group of presented characters is different from the rest. The participant must mark the character that is different as quickly as possible.

Fixation disparity: A red cross and a green arrow were used to measure fixation disparity. Aligning the arrow with the cross, both vertically and horizontally, we obtained the vertical and horizontal disparity of all participants. Participants wore green/red lenses. The obtained deviations were measured, according to distance, in prismatic dioptres. The results of the test include horizontal disparity measured in prismatic dioptres, with an internal or external base depending on the colour selection in each eye during the measurement (the red filter was always used in front of the directing eye), as well as the number of upper base or lower prisms for vertical measurements.

Saccadic eye movements: The character marking test was used. In this test, a set of characters plus a peripheral character are presented. The participant must observe them and mark them saccadicly in a set of central letters. In this task, the response speed is important, and it will be highlighted.

Speed of recognition: The central tachistoscopic test was used. In this test, a set of characters are presented over a period of five minutes, each appearing for a fraction of a second. The participant must identify the largest number of characters as quickly as possible. The failures in identifying the characters and the execution time are noted.

Visual memory: The tic-tac-toe test was used. In this test, a grid divided into nine spaces, with five of these spaces randomly occupied by symbols, is presented to the participant for one-tenth of a second, with several repetitions. The participant must then mark the location of the symbols as quickly as possible.

### 2.2. Statistical Analysis

SPSS 25.0 software (SPSS Inc., Chicago, IL, USA) was used for the statistical analysis. The parametric distribution of the variables was obtained using the Kolmogorov–Smirnov test (*n* > 50), resulting in a non-parametric distribution. The effect size was obtained according to Cohen’s d measure (0.2 indicates a small effect size, 0.5 medium magnitude, and 0.8 indicates a high magnitude effect). Spearman’s test was used to ascertain whether there was a significant association between continuous quantitative variables. The Mann–Whitney U test was used to compare both groups. Furthermore, a cut-off point of *p* ≥ 0.05 was determined in order to assess statistical significance.

### 2.3. Ethical Approval

This study was approved by the ethics committee of the General Directorate for Research and Development (DGID) of the Higher Institute of Education and Sciences (ISEC) in Lisbon (Portugal). The ethical approval number is 01/27052020.

## 3. Results

The total number of participants was 120 (20 elite athletes and 100 non-athletes; d = 0.22), representing a small sample size according to the Cohen’s d measure. The athletic abilities were classified in relation to the state of the athletes’ home provinces. The age range was from 20 to 60 years old (X ± SD: 33.5 ± 8.2; median (IQR): 33 (11)); amongst athletes, the average age was 35.5 ± 10.0 (median (IQR): 34.5 (12.7)), and amongst non-athletes, it was 33.1 ± 7.7 (median (IQR): 33 (10.5)). In regard to gender, 54.2% were males and 45.8% females (athletes: 60.0% males and 40.0% females; non-athletes: 54.2% males and 45.8% females).

### 3.1. Directional Arrows

**Figure 1:**
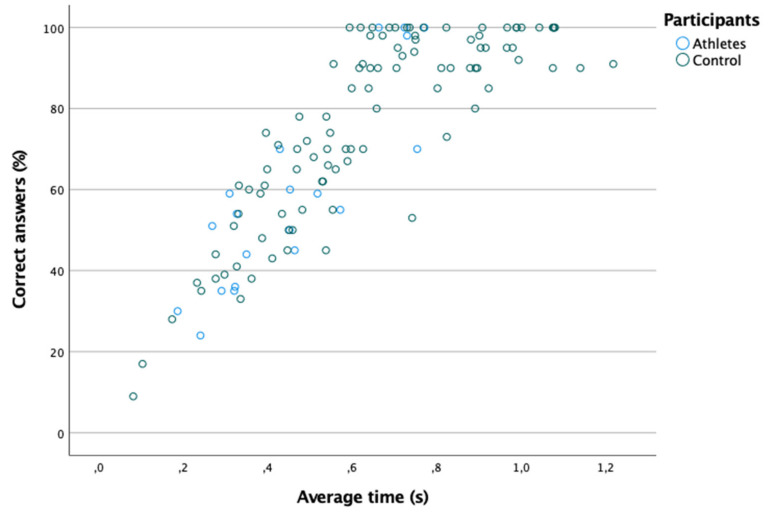
Positive linear relationship between the percentage of correct answers and the average time.

Table 1 shows the percentage of correct answers and the time taken to reach the correct answer in the hand–eye coordination test. Figure 1 shows the existence of a moderate and positive correlation between the average time and the percentage of correct answers (Spearman: rs = 0.845; *p* ≤ 0.001). In turn, significant differences were found in the average time and the percentage of correct answers between the two groups (*p* < 0.05); that is, both the average time and the percentage of correct answers were better amongst athletes (Table 1).

### 3.2. Same Characters

**Figure 2:**
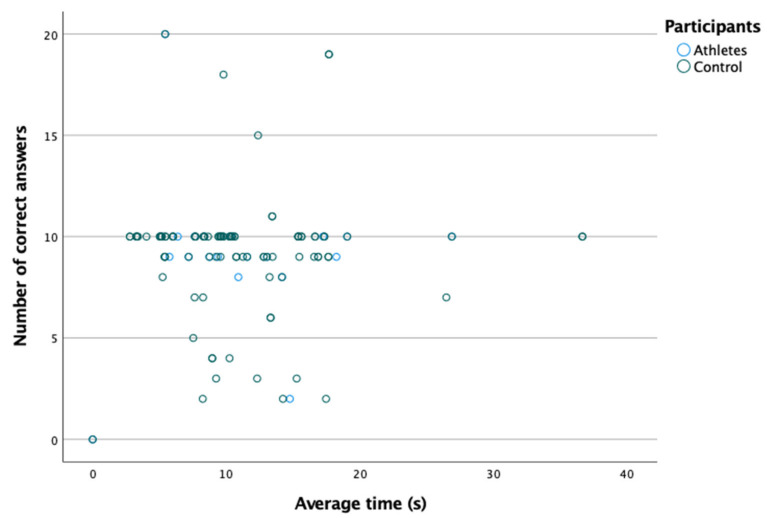
Absence of relationship between the number of correct answers and the average time.

Table 2 shows the number of correct answers and the time taken to reach the correct answer in the peripheral awareness test. As shown in Figure 2, there is no correlation between the average time and the number of correct answers (Spearman: rs= −0.057; *p* = 0.538). Likewise, as shown in Table 2, no significant differences were found in the average time and the number of correct answers between the two groups (*p* > 0.05).

### 3.3. Fixation Disparity

**Figure 3:**
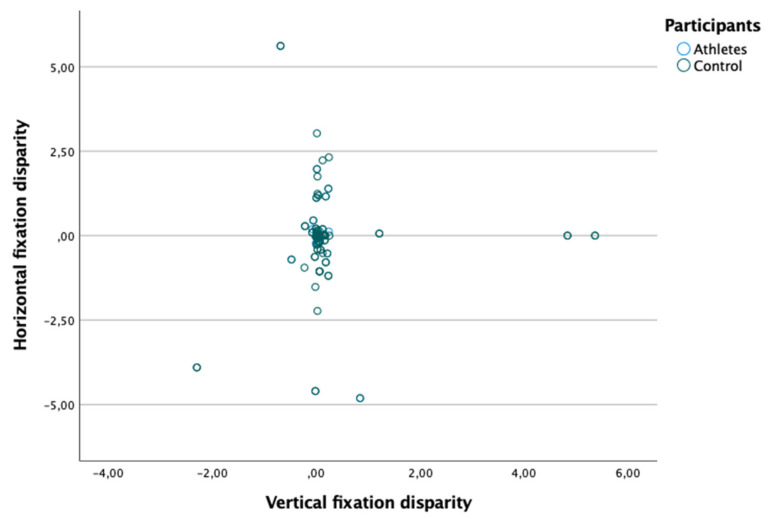
Absence of relationship between the horizontal and vertical fixation disparity.

Table 3 shows the horizontal and vertical fixation disparity. As shown in Figure 3, there is no correlation between horizontal and vertical fixation disparity (Spearman: rs = 0.012; *p* = 0.895). In turn, as shown in Table 3, no significant differences were found in horizontal and vertical fixation disparity between the two groups (*p* > 0.05).

### 3.4. Character Marking

**Figure 4:**
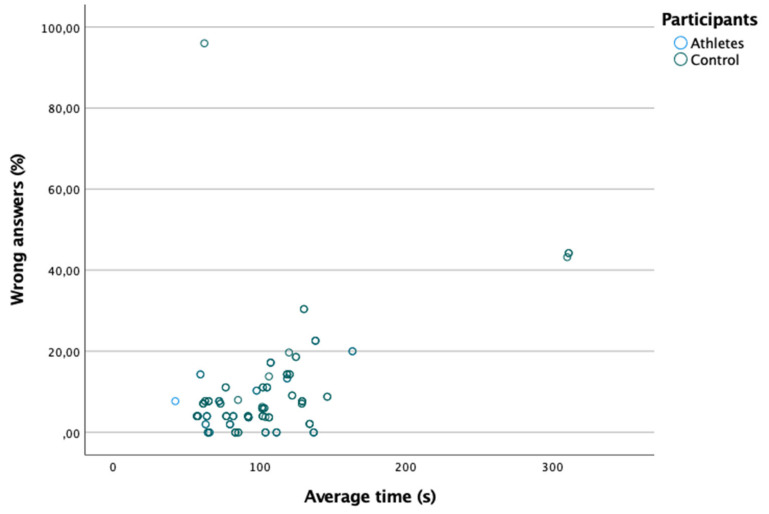
Positive linear relationship between the percentage of wrong answers and the average time.

Table 4 shows the percentage of wrong answers and the time to reach the wrong answer in the saccadic eye movements test. As shown in Figure 4, there is a moderate and positive correlation between the percentage of wrong answers and the average time (Spearman: rs = 0.324; *p* = 0.000). However, no significant differences were found in the percentage of wrong answers and the average time between the two groups (*p* > 0.05) (Table 4).

### 3.5. Central Tachistoscope

**Figure 5:**
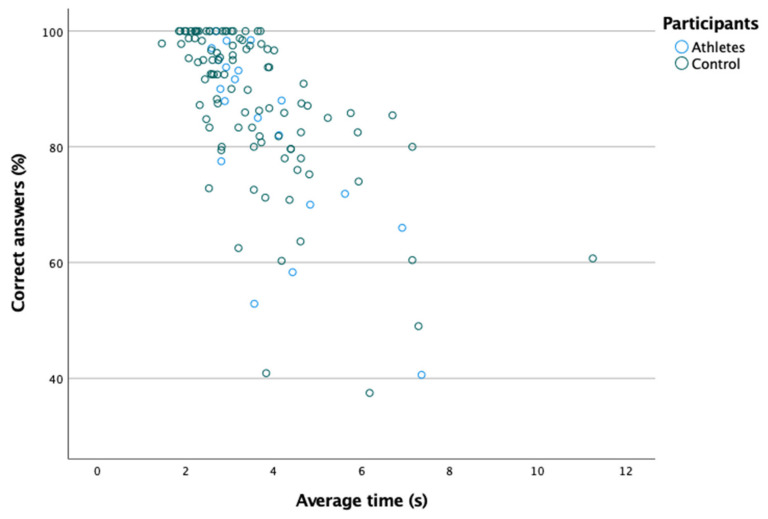
Negative linear relationship between the percentage of correct answers and the average time.

Table 5 shows the percentage of correct answers and the time to reach the correct answer in the speed of recognition test. As shown in Figure 5, a moderate and negative correlation was found between the percentage of correct answers and the average time (Spearman: rs = 0.667; *p* = 0.000). However, as shown in Table 5, no significant differences were found in the percentage of correct answers and the average time between the two groups (*p* > 0.05).

### 3.6. Tic-Tac-Toe

**Figure 6:**
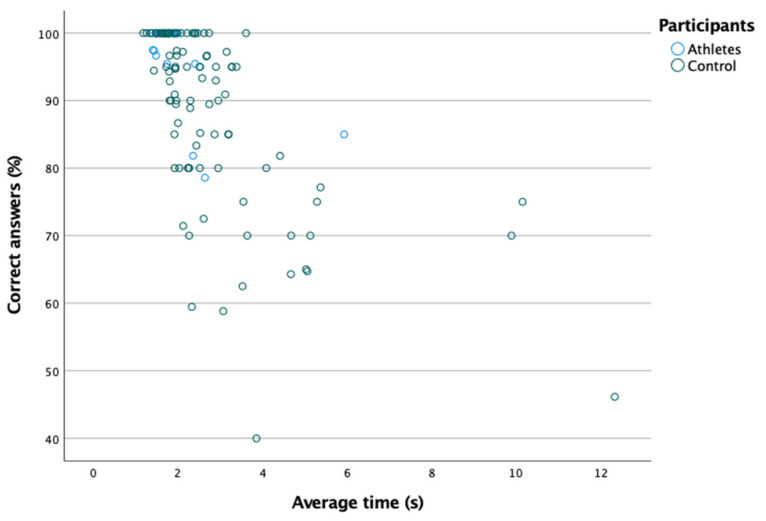
Negative linear relationship between the percentage of correct answers and the average time.

Table 6 shows the percentage of correct answers and the time to reach the correct answer in the visual memory test. Figure 6 shows a moderate and negative correlation between the average time and the percentage of correct answers (Spearman: rs = 0.665; *p* = 0.000). In turn, significant differences were found in the average time and the percentage of correct answers between the two groups (*p* < 0.05); that is, the average time was shorter amongst athletes, and the percentage of correct answers was higher amongst the control group (Table 6).

## 4. Discussion

The objective of this study was to assess a group of skeet shooting athletes and a group of non-athletes with identical and basic visual abilities to verify, through visuospatial intelligence tests, to what extent athletes perform better than non-athletes. The following components were assessed: hand–eye coordination, peripheral awareness, fixation disparity, saccadic eye movements, speed of recognition and visual memory. Only in hand–eye coordination and visual memory did shooters outperform non-athletes.

According to our study, hand–eye coordination results were superior amongst shooters in comparison to non-athletes, as is essential in skeet shooting. There are other sports in which there are no differences, as shown in a study conducted in 2019 concerning hand–eye coordination amongst badminton players, in which the same aforementioned tests were used (athletes and non-athletes had to point at a mark with their finger), resulting in no differences for both groups [41]. In another study conducted in 2017 related to martial arts, athletes outperformed non-athletes in hand–eye coordination tests [42].

Regarding peripheral awareness, unlike other sports, such as football or American football, in which athletes’ results were superior to non-athletes [43], in skeet shooting, no differences were found between the groups.

Fixation disparity is not a test of visuospatial intelligence; however, it is crucial in skeet shooting because of the nature of the sport. Nevertheless, no differences were found between athletes and non-athletes. Under certain circumstances, fixation disparity may indicate differences in oculomotor postural tendencies in relation to gaze position, but the relevance of this in relation to athletes’ performance is arguable [44].

No differences were found between shooters and non-athletes in the saccadic eye movement test as similar studies have noted in relation to other sports. However, there are studies that state that saccadic eye movements are important amongst basketball and baseball players in comparison to non-athletes [45]. In comparison to non-athletes, their athletic peers have a greater ability to resolve spatial dynamic objects during fixation or head movement, as well as the greater ability to focus their attention. Thus, this parameter must be considered for skeet shooting athletes and as an aspect of training [46].

No differences were found in the speed of recognition between athletes and non-athletes in contrast to studies relating to sports such as basketball, volleyball and baseball, in which significant differences were found between athletes and non-athletes [47]. Speed of recognition is inversely proportional to movement and sporting variables; if athletes’ decision-making skills are assessed using tasks that are not specific to their sporting environment, no differences will be found between athletes and non-athletes [48]. Therefore, no differences were found in this test because it must be conducted using shooting-specific test methods; when general test methods are used, the results may be inaccurate.

The visual memory test results showed that athletes were superior in comparison to non-athletes. There are no studies showing that athletes have better visual memory than non-athletes, except for a study on chess players carried out by Chase and Simon in 1973. Their study showed that, when structured scenes were introduced to distinguish between chess masters and beginners, differences were found. However, no differences were found when non-structured scenes were introduced. This also suggests that athletes only perform better with visual memory when experiencing familiar situations. As familiar situations were not used in this study, it is not possible to know how this could have affected the results. Table S1 shows the visual skills required for each sport [49].

Regarding the limitations of our study, currently, there is a paucity of validated software for analysing sports vision. The software used in our study has been used in other published research [50]. COI-SV allows diagnostic tests as well as specific optometric treatments for sports vision and has no distance limitation from the touch screen up to 15 m. In addition, all its programs include various levels of difficulty and allow the user to work by quadrants of the visual field. A further limitation was imposed by the difference in numbers of participants in each group, which resulted in a non-parametric distribution. However, when the assumptions of the nonparametric test were satisfied, they were equally effective. The difference in the sample size was maintained given the value of the effect size. That is, an effect size of 0.2 indicated that the mean of the treated group was at the 58th percentile of the untreated group. Therefore, as the significance of the tests did not change, it was decided to keep the original sample size.

## 5. Conclusions

In recent years, many studies have analysed the visual behaviours of athletes in comparison with those of non-athletes. Many studies in certain disciplines prove that in certain visual skills, elite athletes outperform non-athletes. This study aimed to verify whether for a sport such as skeet shooting, starting from the same basic visual skills, there would be a significant difference regarding specific skills in the six chosen components between athletes and non-athletes. The findings showed that athletes performed only two visual skills better than their non-athletic peers. One of these skills is essential for the sport in question: hand–eye coordination; the other is less important: visual memory. We can conclude that visual expertise tests should be more contextualised with the sport, and, if so, training these abilities can improve the athlete’s sporting performance, once it has been proven that their performance is inferior to that of a non-athlete. In turn, this study confirms that an athlete’s daily sports training and innate qualities pertinent to the practice of this sport do not guarantee that, in terms of vision, their performance will be superior to a non-contact control group.

## Figures and Tables

**Table 1:** “Directional arrows” test results.

	Athletes				Control				
	Mean	SD	Median	CI	Mean	SD	Median	CI	*p*-Value
Percentage of correct answers (%)	58.75	24.23	54.50	47.41–70.09	75.96	22.88	85.00	71.42–80.50	0.007
Time to reach the correct answer (s)	0.46	0.19	0.44	0.37–0.54	0.64	0.25	0.62	0.59–0.69	0.002

SD: standard deviation; CI: confidence interval; s: seconds.

**Table 2:** “Same characters” test results.

	Athletes				Control				
	Mean	SD	Median	CI	Mean	SD	Median	CI	*p*-Value
Number of correct answers	9.05	3.71	9.00	7.32–10.78	9.21	3.10	10.00	8.13–13.87	0.345
Time to reach the correct answer (s)	11.06	6.13	9.85	8.59–9.83	11.15	6.13	10.01	11.15–9.93	0.980

SD: standard deviation; CI: confidence interval; s: seconds.

**Table 3:** Fixation disparity results.

	Athletes				Control				
	Mean	SD	Median	CI	Mean	SD	Median	CI	*p*-Value
Horizontal fixation disparity (arc minutes)	−0.02	1.32	−0.02	−0.04–0.18	−0.09	1.47	−0.005	−0.004–0.42	0.765
Vertical fixation disparity (arc minutes)	0.07	0.23	0.025	−0.63–0.60	0.21	1.08	0.020	−0.38–0.20	0.791

SD: standard deviation; CI: confidence interval.

**Table 4:** “Character marking” test result.

	Athletes				Control				
	Mean	SD	Median	CI	Mean	SD	Median	CI	*p*-Value
Percentage of wrong answers (%)	7.19	7.14	4.00	3.85–10.54	9.42	12.47	7.10	6.94–11.89	0.457
Time to reach the wrong answer (s)	91.24	31.88	87.09	76.32–106.16	103.39	45.01	102.00	94.46–112.32	0.299

SD: standard deviation; CI: confidence interval; s: seconds.

**Table 5:** “Central tachistoscope” test results.

	Athletes				Control				
	Mean	SD	Median	CI	Mean	SD	Median	CI	*p*-Value
Percentage of correct answers (%)	81.88	16.86	87.95	73.99–89.77	88.04	13.27	92.50	85.40–90.67	0.084
Time to reach the correct answer (s)	3.84	1.39	3.34	3.19–4.49	3.51	1.46	3.140	3.22–3.80	0.168

SD: standard deviation; CI: confidence interval; s: seconds.

**Table 6:** “Tic-tac-toe” test results.

	Athletes				Control				
	Mean	SD	Median	CI	Mean	SD	Median	CI	*p*-Value
Percentage of correct answers (%)	96.14	6.52	100.00	93.09–99.19	88.69	13.23	94.59	86.07–91.32	0.008
Time to reach the correct answer (s)	2.06	1.00	1.826	1.59–2.53	2.74	1.72	2.25	2.74–2.40	0.007

SD: standard deviation; CI: confidence interval; s: seconds.

## Data Availability

Not applicable.

## Supplementary Materials

The following are available online at https://www.mdpi.com/article/10.3390/ijerph18158147/s1, Table S1: Visual skills depending on the sport.
