# Supplementary material for: Differences in Visuospatial Expertise between Skeet Shooting Athletes and Non-Athletes
Source: Int J Environ Res Public Health. 2021 Jul 31;18(15):8147. doi: 10.3390/ijerph18158147 (PMC8346005; doi:10.3390/ijerph18158147)
Supplement: Supplementary file 1 [file ijerph-18-08147-s001.zip › ijerph-1305988-Supplementary.pdf]

Supplementary table S1. Visual skills depending on the sport. [International Sports Vision Association (ISVA)] https://www.sportsvision.pro/

Sports	Visual Skills
Baseball/Softball	Eye Tracking and Eye Focusing; Eye Teaming; Anticipation Timing; Visual Attention (Concentration); Depth Perception; Dynamic Visual Acuity; Eye-Hand Coordination; Peripheral Awareness; Visual Reaction Time/Initiated Speed; Visualization
Basketball	Concentration/Focus; Accommodation and Convergence; Anticipation Timing; Depth Perception; Dynamic Visual Acuity; Eye Fatigue and Performance Levels; Fixation Ability; Peripheral Vision/Awareness; Speed and Span of Recognition; Visual Reaction Speed
Boxing	Anticipation Timing; Concentration; Depth Perception; Eye-Hand/Body/Foot Coordination; Eye Fatigue; Eye Motility; Peripheral Vision and Awareness; Speed of Recognition; Visualization
Football	• Quarterbacks: Anticipation Timing; Concentration; Depth Perception; Divergence; Eye-Hand/Foot/Body Coordination; Peripheral Vision/Awareness; Speed and Span of Recognition<br>• Receivers: Accommodation & Convergence; Concentration; Depth Perception; Eye Fatigue; Peripheral Vision/Awareness.<br>• Running Backs: Accommodation & Convergence; Concentration; Eye-Hand Coordination.<br>• Defensive Backs and Safeties: Accommodation & Convergence; Anticipation Timing; Concentration; Eye-Hand Coordination; Peripheral Vision/Awareness<br>• Linebackers: Concentration; Peripheral Vision and Awareness; Speed & Span of Recognition<br>• Kickers: Concentration; Anticipation Timing; Accommodation & Convergence; Depth Perception; Eye-Hand/Body/Foot Coordination
Golf	Concentration; Depth Perception; Eye-Hand/Foot/Body Coordination; Fixation Ability; Focusing and Tracking; Peripheral Awareness; Visualization
Hockey	Accommodation & Convergence; Anticipation Timing; Concentration; Depth Perception; Eye Fatigue and Performance Levels; Eye-Hand Coordination; Peripheral Vision/Awareness; Visual Reaction Time
Motorsports	Accommodation and Convergence; Anticipation Timing; Concentration; Depth Perception; Dynamic Visual Acuity; Eye-Hand Coordination/The Visual System Leads The Motor System; Peripheral Awareness; Speed and Span of Recognition
Skiing	Accommodation and Convergence; Anticipation Timing; Concentration; Depth Perception; Eye-Hand/Foot/Body

	Coordination; Peripheral Awareness; Speed and Span of Recognition; Visual Reaction Speed/Time
Soccer	Accommodation and Convergence; Concentration; Depth Perception; Eye Fatigue; Eye-Hand/Body/Foot Coordination; Fixation Ability; Peripheral Vision/Awareness; Speed and Span of Recognition
Table Tennis	Accommodation and Convergence; Anticipation Timing; Concentration; Depth Perception; Eye Fatigue; Eye-Hand Coordination; Peripheral Vision/Awareness; Speed and Span of Recognition; Visual Reaction Time/Speed
Tennis	Accommodation & Convergence; Anticipation Timing; Concentration; Depth Perception; Eye Fatigue; Eye-Hand Coordination; Peripheral Vision/Awareness; Speed and Span of Recognition
